# Editorial: Use of digital human modeling for promoting health, care and well‐being

**DOI:** 10.3389/fbioe.2025.1731836

**Published:** 2026-01-30

**Authors:** Sofia Scataglini, Ravindra S. Goonetilleke, Steven Truijen

**Affiliations:** 1 4D4ALL Lab, Department of Rehabilitation Sciences and Physiotherapy, Center for Health and Technology (CHaT), Faculty of Medicine and Health Sciences, University of Antwerp, Antwerp, Belgium; 2 Department of Management Science and Engineering, Khalifa University, Abu Dhabi, United Arab Emirates

**Keywords:** digital human model, digital human modeling (DHM), health, care, well-being

Digital Human Modeling (DHM) has emerged as a transformative model across disciplines, enabling simulation and analysis of human interaction with environments, systems, and products (Scataglini et al., 2019). Through virtual representations of the human body, DHM allows clinicians, designers, and engineers to explore complex physiological (Chen et al.), biomechanical (Zerdzicki, et al.), and behavioral phenomena in a controlled digital space. This editorial synthesizes findings from 17 recent studies, highlighting the breadth and depth of DHM applications in ergonomics, rehabilitation, diagnostics (Kim et al.) and safety.

A study using computational fluid dynamics (CFD) developed a thermoregulation model based on 3D human scans to simulate heat transfer in cold environments (Yu et al.). By segmenting the body into core, skin, and clothing layers, it accurately predicted skin and garment temperatures, offering valuable insights for designing protective clothing and improving thermal comfort.

Another investigation focused on postural control, applying rambling-trembling decomposition of center-of-pressure data to evaluate balance under simulated somatosensory deficits (Gerber et al.). Increased sway—especially in the anterior-posterior direction—was observed under sensory disruption, highlighting DHM’s potential in diagnosing balance impairments and preventing falls among older adults.

In rehabilitation, a novel contactless approach used laser displacement sensors to capture free oscillations of atrophic muscles post-ACL surgery (Tomaszewska et al.). Vibration frequency analysis tracked recovery in the rectus femoris, showing convergence with healthy reference values over time, and demonstrating a non-invasive method for monitoring the progress of muscle rehabilitation.

In diagnostics, an innovative RcdNet deep learning model—augmented with attention mechanisms—achieved 93.5% accuracy in classifying benign and malignant breast tumors in ultrasound images (Liu et al.). Heatmap visualizations aligned with radiologists’ focal areas, showing DHM’s potential to support clinical decisions and reduce diagnostic variability.

DHM also supports personalized medicine. A hybrid neural network combining Temporal Convolutional Networks and LSTM layers predicted Cobb angle progression in adolescents with idiopathic scoliosis undergoing Schroth therapy (Yin et al.). Based on surface EMG signals, the model provided real-time feedback, enabling personalized treatment strategies.

In trauma modeling, a study reconstructed 72 pedestrian collisions using finite element head models and wavelet packet energy analysis to define injury risk functions for repetitive traumatic brain injury (Xiong et al.). It revealed distinct thresholds for repetitive versus single impacts, informing updated safety standards in automotive and sports contexts.

Extended reality (XR) technologies are increasingly paired with DHM in rehabilitation (Lu et al.). A systematic review of 16 studies found that XR integrated with 3D/4D DHM improved physical, psychological, and metabolic outcomes in populations with stroke, diabetes, and eating disorders. Despite methodological variation, XR-DHM systems show strong potential for immersive and adaptive therapy.

In orthopedic biomechanics, finite element models assessed stress distribution in intertrochanteric fractures treated with intramedullary nails (Zhu et al.). Positive medial cortical support reduced stress and displacement, suggesting it as a viable alternative to anatomical reduction. Another study evaluated femoral head collapse risk post-fixation removal, finding that bone grafting improved biomechanical behavior and may reduce osteonecrosis risk (Li et al.).

DHM also advances neuromusculoskeletal modeling. A CNN-GRU-Attention model, enhanced with transfer learning, predicted knee joint torque using EMG and kinematic data (Xie et al.). It showed low error rates and high cross-subject generalizability, supporting applications in injury prevention and motion analysis.

In spinal biomechanics, simulations of multifidus muscle atrophy revealed increased stress in cervical discs, joint capsules, and cartilage endplates—especially in lower segments—highlighting the importance of targeted exercise in preventing chronic neck pain (Xu et al.). Another study compared cortical bone trajectory screws with traditional pedicle screws in lumbar fixation (Li et al.). Cortical screws offered better stability but generated higher stress in osteoporotic bone, guiding surgical choices based on patient bone quality.

DHM is also employed in the prediction of motion. A deep learning model trained on DHM data accurately predicted human limb trajectories during dynamic tasks, with applications in robotics, prosthetics, and human-computer interaction. In occupational health, DHM combined with sensor data analyzed driver posture and fatigue in long-haul driving (Ando et al.). The model identified deviations and fatigue markers, suggesting ergonomic interventions to reduce strain and improve safety.

Finally, DHM was applied to simulate worker interactions in industrial workstations. By modeling reach, posture, and exertion, researchers optimized layout and tool placement, reducing injury risks while enhancing productivity.

These studies demonstrate the wide-ranging impact and interdisciplinary reach of digital human modeling. From diagnostics and rehabilitation to workplace safety and personalized medicine, DHM is reshaping how we understand and support human health and performance. Its integration with artificial intelligence, extended reality, and biomechanical simulation continues to unlock new frontiers in precision design, clinical care, and injury prevention.

Looking ahead, future research should focus on standardizing DHM methodologies, validating models across diverse populations, and incorporating real-time data for adaptive interventions.

The true potential of Digital Human Modeling (DHM) extends beyond the mere simulation of the human body; it lies in its capacity to enhance our understanding of human biomechanics, behavior, and interaction within digital environments. By enabling safe, controlled, and inclusive experimentation, DHM facilitates the development of ergonomic designs, personalized healthcare solutions (Danckaers et al.), and adaptive technologies that account for diverse human needs and capabilities.

## References

[B1] Scataglini S. Paul G. (2019). DHM and Posturography. Academic Press.

